# Characterization of the left ventricular arrhythmogenic substrate with multimodality imaging: role of innervation imaging and left ventricular global longitudinal strain

**DOI:** 10.1186/s41824-019-0060-8

**Published:** 2019-08-02

**Authors:** Mohammed El Mahdiui, Jeff M. Smit, Alexander R. van Rosendael, Victoria Delgado, Nina Ajmone Marsan, J. Wouter Jukema, Arthur J. H. A. Scholte, Jeroen J. Bax

**Affiliations:** Department of Cardiology, Heart Lung Centre, Leiden University Medical Centre, Albinusdreef 2, 2300 RC Leiden, The Netherlands

**Keywords:** Global longitudinal strain, ^123^I-meta-iodobenzylguanidine, Sudden cardiac death, Arrhythmogenic substrate

## Abstract

**Background:**

Even though implantable cardioverter defibrillator (ICD) implantation for primary prevention has shown to reduce the risk of sudden cardiac death in chronic heart failure patients with reduced left ventricular ejection fraction (LVEF), a significant portion of these patients will never receive appropriate ICD therapy. We aimed to functionally characterize the arrhythmogenic substrate using left ventricular (LV) global longitudinal strain (GLS) and heart-to-mediastinum (H/M) ratio on ^123^I-meta-iodobenzylguanidine (^123^I-MIBG) scintigraphy.

**Methods:**

We included patients with heart failure with reduced LVEF who received an ICD for primary prevention. To functionally characterize the arrhythmogenic substrate, we measured the LV GLS with two-dimensional speckle tracking echocardiography and cardiac innervation measured as the H/M ratio on ^123^I-MIBG scintigraphy. An event was defined as appropriate ICD therapy.

**Results:**

A total of 155 patients were included, 74% were male and the mean age was 72 ± 9 years. During a median follow-up of 10 (6–12) years, 43 patients (28%) experienced appropriate ICD therapy. Patients that experienced an event were more often male, had more often ischaemic cardiomyopathy and were more likely to have worse renal function. There was no difference in the left ventricular ejection fraction (LVEF) between the two groups (25 ± 6.4% vs 26 ± 6.0%, *p* = 0.276). However, LV GLS was significantly more impaired in the group that experienced an event compared to patients that did not (− 6.7 ± 2.1% vs − 7.6 ± 2.1%; *p* = 0.020). The innervation, measured as the H/M ratio on ^123^I-MIBG scintigraphy was also significantly more impaired in the patients that experienced and event compared to patients that did not (1.34 ± 0.2 vs 1.47 ± 0.2, *p* ≤ 0.001). Multivariable Cox regression analysis showed LV GLS and H/M ratio independently associated with appropriate ICD therapy with a hazard ratio of 1.24 (95% CI 1.027–1.491, *p* = 0.025) and 5.71 (95% CI 1.135–28.571, *p* = 0.034), respectively. LV GLS and H/M ratio were significantly correlated (Pearson correlation coefficient − 0.30, *p* < 0.001).

**Conclusions:**

Functionally characterizing the arrhythmogenic substrate using different imaging techniques defines the risk for appropriate ICD therapy, whereas LVEF did not.

## Background

Evaluation of heart failure patients who may benefit from implantable cardioverter defibrillator (ICD) remains challenging. Current guidelines recommend an ICD implantation as primary prevention in symptomatic heart failure patients with a left ventricular ejection fraction (LVEF) ≤ 35% treated with optimal medical therapy (Priori et al. 2015). However, around 40% of these patients receiving an ICD will not develop ventricular arrhythmias that can be appropriately treated with the ICD (Moss et al. 2004). Therefore, a more individualized risk stratification strategy is necessary.

Characterization of the anatomical and functional substrate that may lead to re-entrant ventricular tachycardia and sudden cardiac death has been shown feasible with various imaging techniques (Bertini et al. 2012). Anatomically, the arrhythmogenic substrate is characterized by bundles of scar, fibrous tissue intermingled with viable myocardium (Fernandez-Armenta et al. 2013; Lin et al. 2013). Functionally, the arrhythmogenic substrate is characterized by heterogeneous regional contraction and denervated myocardium (Boogers et al. 2010; Ersboll et al. 2013; Jacobson et al. 2010). Transient factors, such as ischaemia, can influence this arrhythmogenic substrate and serve as trigger for re-entry tachyarrhythmias. Left ventricular (LV) global longitudinal strain (GLS) measured with two-dimensional (2D) speckle tracking echocardiography describes better than the LVEF heterogeneous contraction of the LV myocardium and has been associated with LV myocardial scar burden (Bello et al. 2005; Gulati et al. 2013; Schmidt et al. 2007). In addition, in ischaemic heart failure, the value of longitudinal strain of the peri-infarct zone has been associated with appropriate ICD therapy (Ng et al. 2010). Sympathetic myocardial innervation can be assessed with ^123^I-meta-iodobenzylguanidine (^123^I-MIBG) scintigraphy. This radiotracer is an analogue of norepinephrine up taken by the sympathetic nerve terminals of the heart, without being metabolized. In heart failure patients, the uptake of ^123^I-MIBG by the heart is reduced, indicating myocardial denervation. A reduced heart-to-mediastinum (H/M) ratio of the ^123^I-MIBG uptake on planar imaging has been associated with increased risk of ventricular arrhythmic events (Jacobson et al. 2010; Nagahara et al. 2008). So far, there is no study using multimodality imaging to functionally assess the arrhythmogenic substrate.

The aim of the present study was to functionally characterize the arrhythmogenic substrate of heart failure patients receiving an ICD by measuring LV GLS and the H/M ratio on planar ^123^I-MIBG scintigraphy imaging and study the differences between patients presenting with appropriate ICD therapies and patients without.

## Methods

### Patients and clinical evaluation

Heart failure patients who received an ICD for primary prevention, according to prevailing guidelines (Priori et al. 2015), and who underwent clinically indicated a ^123^I-MIBG scintigraphy between 2004 and 2017 at the Leiden University Medical Centre (The Netherlands) were retrospectively evaluated. Demographic and clinical characteristics were collected using the departmental cardiology information system (EPD-vision; Leiden University Medical Centre, Leiden, The Netherlands) and electronic medical records (HiX; ChipSoft, Amsterdam, The Netherlands) and retrospectively analysed. Echocardiographic data were stored in the echocardiographic database and the planar ^123^I-MIBG scintigraphy data in the department of nuclear medicine database. Clinical, echocardiographic and ^123^I-MIBG scintigraphy data were analysed retrospectively. The association between clinical, conventional and advanced (2D speckle tracking imaging) echocardiographic variables and ^123^I-MIBG variables and ventricular arrhythmic events was investigated. For retrospective analysis of clinically acquired data anonymously handled, the institutional review board waived the need for written patient informed consent.

### Transthoracic echocardiography: conventional and speckle tracking analysis

Transthoracic echocardiography was performed with the patients at rest, in the left lateral decubitus position using a commercially available system (Vivid 7 and E9, GE Healthcare, Horten, Norway). Bimodal, M-mode, colour, continuous and pulsed wave Doppler data were acquired with 3.5 MHz or M5S transducers and digitally stored in cine-loop format. Offline analysis was performed using the EchoPac system (version BT13, GE Medical Systems, Horten, Norway). Left ventricular end-diastolic volume (LVEDV) and left ventricular end-systolic volume (LVESV) were measured on the apical two- and four-chamber views according to the Simpson biplane method and the LVEF was calculated (Lang et al. 2015).

Using 2D speckle tracking echocardiography, the LV GLS was measured. After manually tracing the endocardial border of the left ventricle in the long-axis, two- and four-chamber views, the regions of interest (ROI) were automatically created and adjusted to the thickness of the myocardium. The software automatically tracks the myocardium throughout the cardiac cycle and the quality of the tracking is evaluated. The LV GLS was calculated as the average of the peak systolic longitudinal strain of the three apical views and the results were displayed in a 17-segment “bull’s-eye” plot.

### ^123^I-MIBG scintigraphy data acquisition and analysis

For assessment of myocardial innervation, 185 MBq of ^123^I-MIBG (AdreView, GE Healthcare, Princeton, NJ, USA) was injected intravenously, after thyroid blockage with sodium iodide. Using a dual-head gamma camera (GCA-7200, Toshiba Corp., Tokyo, Japan) equipped with a low-energy high-resolution collimators, planar anterior images of the thorax were obtained 4 h (late) after tracer injection. Images were obtained with a 15% energy window centred at the 159 keV energy peak of 123I and were subsequently stored in a 256 × 256 matrix.

A ROI was manually drawn over the entire heart to measure the counts in the LV myocardial region. To measure the counts in the mediastinal region, a rectangular ROI was placed on the upper half of the mediastinum using the lung apex, upper cardiac border and medial contours of the lungs as borders. The late H/M ratio was calculated by dividing the mean counts in the cardiac ROI by the mean counts in the mediastinal ROI. The analysis was performed using dedicated post-processing software on a Syngo-MI workstation (Siemens Medical Solutions, Malvern, PA, USA).

### Follow-up

Patients were followed up at the heart failure outpatient clinic and the ICD devices were interrogated on 6-monthly visits. The ICD ventricular tachyarrhythmia detection criteria and therapy were programmed conventionally. The occurrence of appropriate ICD therapy was the primary endpoint. Appropriate ICD therapy was defined as anti-tachycardia pacing or shock for ventricular tachycardia or ventricular fibrillation.

### Statistical analysis

Continuous variables are reported as mean ± standard deviation if normally distributed, and as median and 25–75% interquartile range (IQR) if non-normally distributed. Categorical data are presented as frequencies and percentages. Patients were divided in two groups according to the occurrence of an event, appropriate ICD therapy or not. Continuous data were compared between patients presenting with event and patients without event using the Student’s *t* test or the Mann-Whitney *U* test. Categorical data were compared with the *χ*2 test. Cox proportional hazard regression analyses were used to evaluate the variables that were significantly associated with appropriate ICD therapy. The relationship between LV GLS and H/M ratio was investigated using the Pearson correlation. A *p* value < 0.05 was considered statistically significant. All statistical analyses were performed using the SPSS software package (IBM Corp. Released 2015, IBM SPSS Statistics for Windows, Version 23.0. Armonk, NY, USA: IBM Corp.).

## Results

A total of 155 patients with ^123^I-MIBG scintigraphy and echocardiography with LV GLS data were included (74% were male and the mean age was 72 ± 9 years). During a median follow-up of 10 years (25–75% IQR 6–12 years), appropriate ICD therapy occurred in 43 patients (28%). An ICD shock was the first appropriate ICD therapy in 70% of the cases. Table 1 shows the baseline clinical characteristics for the total population and the patients who did or did not experience an appropriate ICD therapy. The majority of patients referred had New York Heart Association (NYHA) functional class II or III heart failure symptoms. The two groups of patients were comparable in various demographic and clinical characteristics.

**Table 1:** Patient clinical characteristics

	All Patients (*n* = 155)	Patients without appropriate ICD therapy (*n* = 112)	Patients with appropriate ICD therapy (*n* = 43)	*p*-value
Clinical characteristics				
Age	72 ± 8.9	72 ± 8.7	71 ± 9.3	0.700
Male, n(%)	115 (74)	79 (71)	36 (84)	0.093
NYHA functional class, n(%)				
I	21 (14)	16 (14)	5 (12)	0.817
II	51 (33)	35 (31)	16 (37)	
III/IV	80 (52)	59 (53)	21 (49)	
Ischemic cardiomyopathy, n(%)	88 (57)	60 (54)	28 (65)	0.194
Devices				
ICD, n(%)	51 (33)	36 (32)	15 (35)	0.745
CRT-D, n(%)	104 (67)	76 (68)	28 (65)	
Cardiovascular risk factors				
Dyslipidemia, n(%)	44 (28)	30 (27)	14 (33)	0.475
Diabetes, n(%)	25 (16)	19 (17)	6 (14)	0.648
Hypertension, n(%)	57 (37)	41 (37)	16 (37)	0.945
Smoking, n(%)	35 (23)	25 (22)	10 (23)	0.901
Medication				
ACE-I/ARB’s, n(%)	132 (85)	97 (87)	35 (81)	0.414
Diuretics, n(%)	115 (74)	79 (71)	36 (84)	0.093
Beta-blockers, n(%)	118 (76)	83 (74)	35 (81)	0.341
-Sotalol	5 (4)	4 (4)	1 (2)	1.000
Calcium-blockers, n(%)	77 (50)	55 (49)	22 (51)	0.819
Amiodarone, n(%)	30 (19)	23 (21)	7 (16)	0.548
Digoxin, n(%)	17 (11)	14 (13)	3 (7)	0.325
Statin, n(%)	97 (63)	70 (63)	27 (63)	0.973
Anti-diabetic medication, n(%)	83 (54)	58 (52)	25 (58)	0.478
Laboratory results				
Creatinine (mmol/L)	101 ± 32	100 ± 31	105 ± 35	0.368
Hematocrit (%)	41.6 ± 3.3	41.6 ± 3.3	41.5 ± 3.5	0.887

Values are mean ± standard deviation or n (%).

*ACE-I* Angiotensin converting enzyme inhibitor, *ARB’s* Angiotensin II receptor blockers, *CRT-D* Cardiac resynchronization therapy defibrillator, *ICD* Implantable cardioverter defibrillator, *NYHA* The New York Heart Association.

**Fig. 1:**
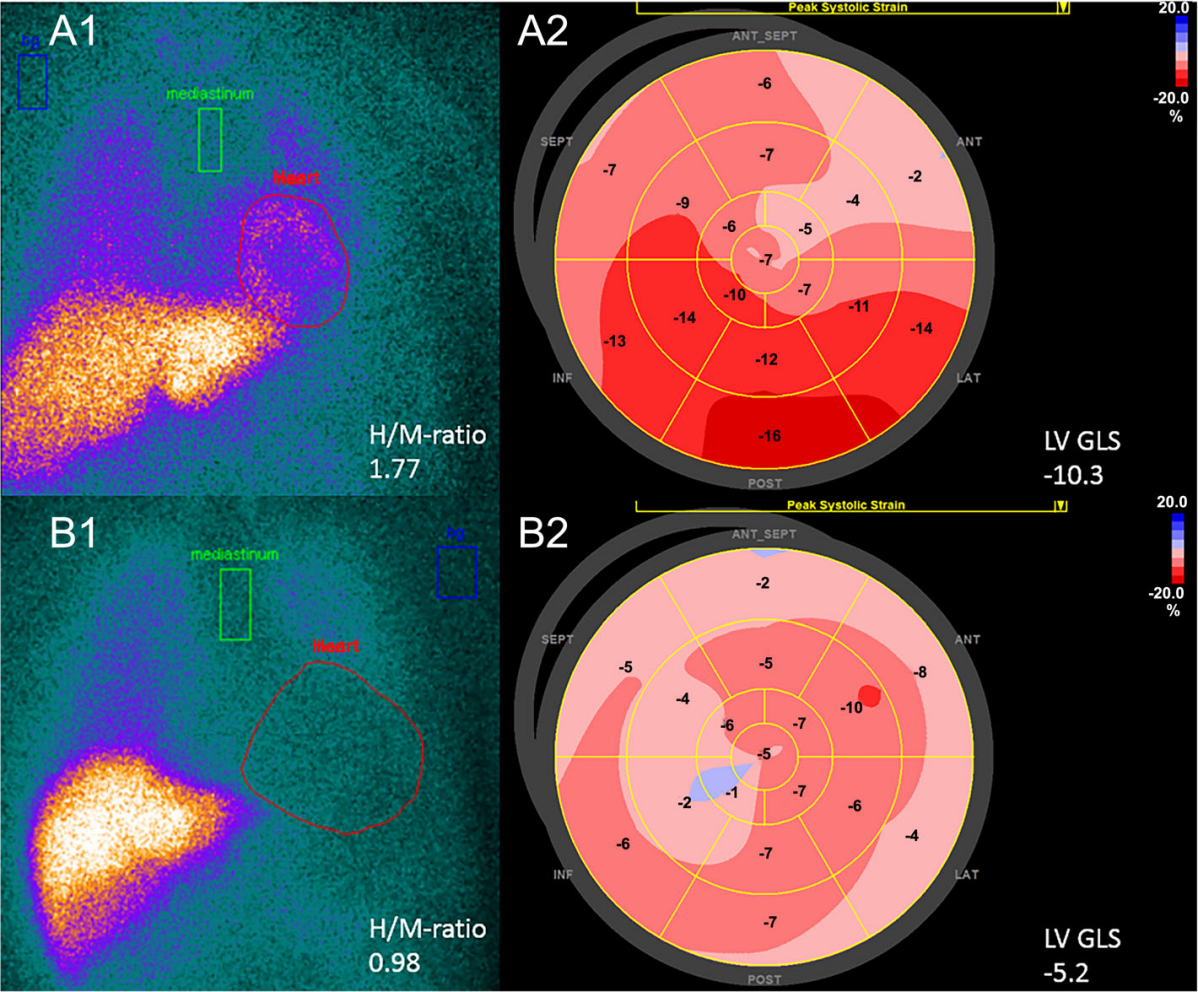
Example of H/M-ratio and LV GLS. Example of heart-to-mediastinum (H/M) ratio on planar images (**A1** and **B1**) and left ventricular (LV) global longitudinal strain (GLS) (**A2** and **B2**) of two patients. Patient A, 70-year-old male, with ischemic cardiomyopathy, left ventricular ejection fraction (LVEF) 34%. Patient B, 72-year-old male, with ischaemic cardiomyopathy, LVEF 25%. Patient A did not experience an event, whereas patient B experienced appropriate ICD therapy

The imaging characteristics are shown in Table 2. Patients with an event had significantly larger LVEDV as compared to patients without an event. However, the groups were comparable in terms of LVEF (25 ± 6.4% vs 26 ± 6.0%; *p* = 0.276). In contrast, patients who presented with an event showed significantly more impaired LV GLS compared to their counterparts (− 6.7 ± 2.1% vs − 7.6 ± 2.1%; *p* = 0.020, Fig. 1). In the planar ^123^I-MIBG data, the late H/M ratio was significantly lower in the group of patients who experienced an event compared to the group who did not (1.34 ± 0.2 vs 1.47 ± 0.2, *p* ≤ 0.001, Fig. 1). Multivariate Cox regression analysis showed that LV GLS and H/M ratio were independently associated with appropriate ICD therapy, with a hazard ratio of 1.24 (95% CI 1.027–1.491, *p* = 0.025) for LV GLS and 5.71 (95% CI 1.135–28.571, *p* = 0.034) for H/M ratio when corrected for LVEDV and LVEF.

**Table 2:** LV GLS and ^123^I-MIBG characteristics

	All Patients (*n* = 155)	Patients without appropriate ICD therapy (*n* = 112)	Patients with appropriate ICD therapy (*n* = 43)	*p*-value
Two-dimensional echocardiography				
Left ventricular end-diastolic volume, (ml)	247 ± 87	236 ± 83	274 ± 90	0.017
Left ventricular end- systolic volume, (ml)	186 ± 72	181 ± 71	201 ± 73	0.117
Left ventricular ejection fraction, (%)	25 ± 6.3	25 ± 6.4	26 ± 6.0	0.276
Left ventricular GLS, (%)	−7.4 ± 2.2	−7.6 ± 2.1	−6.7 ± 2.2	0.020
^123^I-MIBG SPECT				
Heart mediastinum ratio late	1.43 ± 0.2	1.47 ± 0.2	1.34 ± 0.2	<0.001

Values are mean ± standard deviation

*GLS* Global longitudinal strain

**Fig. 2:**
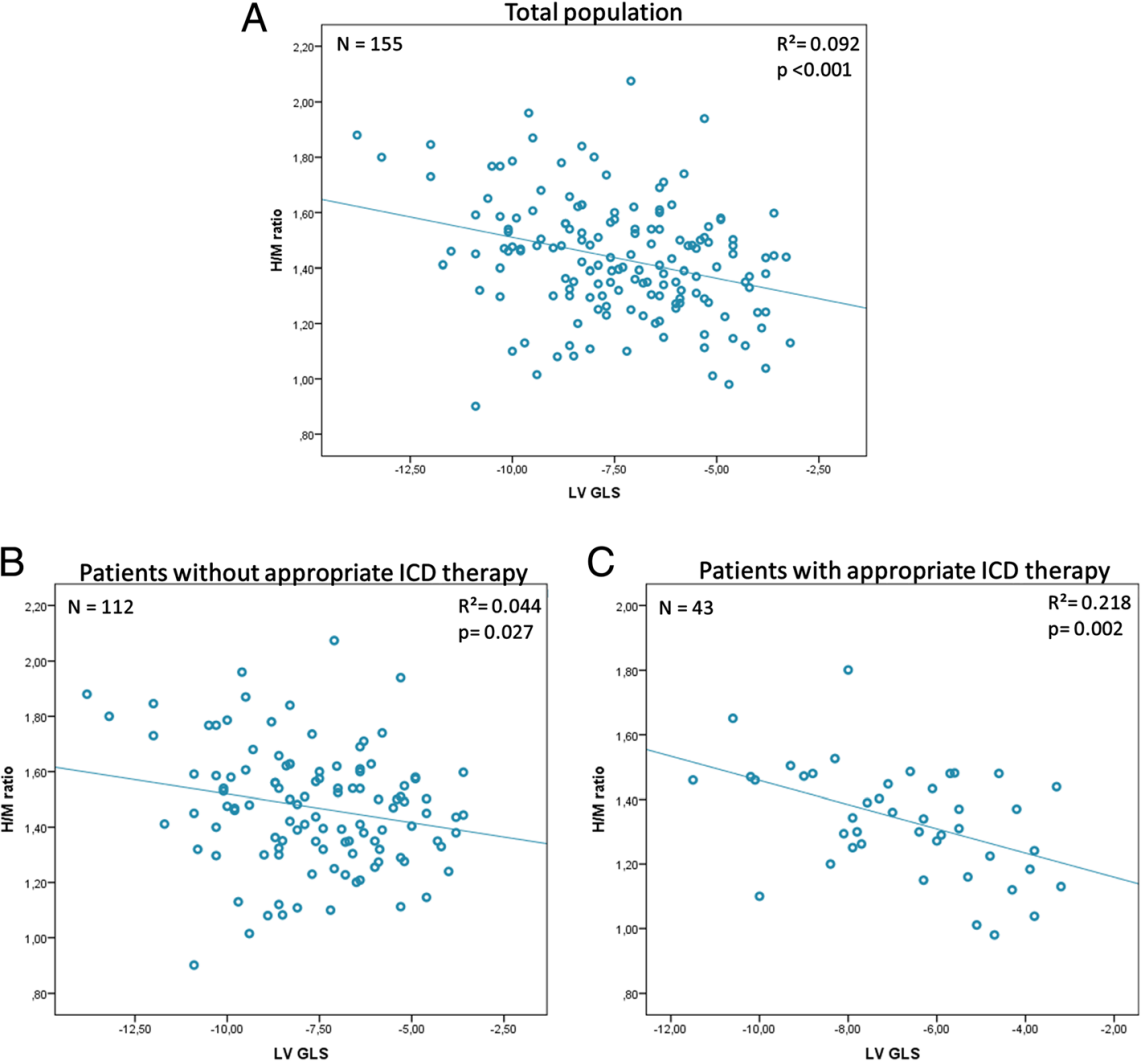
Correlation between H/M ratio and LV GLS. Correlation between heart-to-mediastinum (H/M) ratio and left ventricular global longitudinal strain (LV GLS) in the total population (**a**), patients who did not experience an event (**b**) and patients who did experience an event (**c**). There was a significant correlation between H/M ratio and LV GLS using the Pearson correlation coefficient for the total population (*r* = 0.30, *p* < 0.001), patients who did not experience an event (*r* = − 0.21, *p* = 0.027) and those who did (*r* = − 0.50, *p* = 0.002)

There was a moderate but significant correlation between LV GLS and H/M ratio (*r* = 0.30, *p* < 0.001, Fig. 2). This correlation remained significant in the group that did not experience an event (*r* = − 0.21, *p* = 0.027) and was stronger in the group that did experience an event (*r* = − 0.50, *p* = 0.002) (Fig. 2).

## Discussion

Despite showing similar LVEF, heart failure patients experiencing appropriate ICD therapy showed more impaired LV systolic function when measured with 2D speckle tracking GLS and more impaired myocardial innervation as assessed with ^123^I-MIBG scintigraphy. These results provide additional evidence on the limited value of LVEF to identify heart failure patients who will benefit from an ICD.

### Assessment of arrhythmogenic substrate

The structural LV changes that underlie heart failure with reduced LVEF include expansion of extracellular matrix with increase of collagen and fibroblasts. Scar or fibrotic tissues (bundles of collagen) are not per se pro-arrhythmic since they are electrically inert tissue. However, the areas where the fibrous/scar tissue intermingles with viable myocytes create a substrate where re-entry circuits may form and lead to ventricular arrhythmias (Morita et al. 2014). After myocardial infarction, this area is known as border zone.

The border zone can be characterized using different imaging modalities. On late gadolinium contrast enhanced cardiovascular magnetic resonance (LGE CMR), the border zone shows intermediate signal intensity between normal myocardium and dense myocardial scar. Applying specific thresholds, the amount of border zone can be quantified. In 91 patients with ischaemic heart disease and indication for ICD implantation as primary prevention, Roes et al. demonstrated that each 10-g increase in the mass of border zone was associated with 51% increased risk of ventricular arrhythmias and appropriate ICD shocks (Roes et al. 2009a). The functional properties of the scar zone, border zone and normal myocardium can be evaluated with 2-dimensional speckle tracking echocardiography and the measurement of LV regional longitudinal strain. In ischaemic heart failure, LV segments with values of longitudinal strain more impaired than − 4.5% (less negative) have been shown to correspond to areas of transmural scar (sensitivity 81.2% and specificity 81.6%) (Roes et al. 2009b). The areas surrounding the LV segments with a value of longitudinal strain more negative than − 4.5% can be considered border zone. In 424 ischaemic heart failure patients who received an ICD as primary prevention, Ng et al. showed that the occurrence of ventricular arrhythmias and appropriate ICD shocks during follow-up was more frequent as the longitudinal strain of the border zone was more impaired (hazard ratio 1.25) (Ng et al. 2010). The present study provides additional evidence on the association between impaired LV GLS and the occurrence of appropriate ICD therapies. More impaired LV GLS reflects a larger burden of myocardial fibrosis which may lead to areas of slow conduction and conduction block and favour the formation of re-entry circuits and ventricular arrhythmias. In addition, the association between LV GLS and sudden cardiac death, ventricular arrhythmias or appropriate ICD therapy has been also demonstrated in previous studies including 988 patients with acute myocardial infarction and LVEF > 35% in 91% of them (Ersboll et al. 2013). On multivariate analysis, LV GLS was independently associated with the occurrence of sudden cardiac death or ventricular arrhythmias with a hazard ratio of 1.24, similar to the hazard ratio reported in the current study.

Moreover, the border zone is also characterized by viable myocardium with abnormal innervation. Previous studies have shown that in ischaemic cardiomyopathy patients, the areas of denervation exceed the area of perfusion defects on single photon emission tomography (SPECT) (Bax et al. 2008; Gimelli et al. 2014; Matsunari et al. 2000). The higher susceptibility of sympathetic nerve fibres to ischaemia compared to myocytes leads to larger areas of denervation than scar after myocardial infarction (denervation-perfusion mismatch) (Matsunari et al. 2000; McGhie et al. 1991; Zipes 1990). By assessing myocardial denervation with ^123^I-MIBG SPECT and myocardial perfusion with ^99m^Technetium-tetrofosmin SPECT, the mismatch can be quantified. Patients with larger areas of denervation-perfusion mismatch have shown a higher incidence of ventricular arrhythmia events (Boogers et al. 2010). Using planar ^123^I-MIBG scintigraphy, the AdreView Myocardial Imaging for Risk Evaluation in Heart Failure (ADMIRE-HF) study, the largest study so far including 964 heart failure patients followed for 2 years, showed that patients with an H/M ratio between 1.2 and 1.6 had more frequent ventricular arrhythmic events as compared to patients with an H/M ratio ≥ 1.6 (Jacobson et al. 2010). This parameter reflects the global cardiac sympathetic innervation rather than characterizing the border zone where the re-entrant ventricular arrhythmias may start. In the present study, patients who received appropriate ICD therapy showed more impaired H/M ratio compared to those who remain without event.

The correlation between LV GLS and H/M ratio was moderate, indicating the distinct differences between both modalities. Our findings are in agreement with previously published studies, reporting similar correlations between LV GLS and H/M ratio (Bulten et al. 2015; Cruz et al. 2019). In clinical practice, a combination of multimodality imaging to characterize the arrhythmogenic substrate from the anatomical and functional point of view may be important to accurately identify the patients who will benefit from an ICD implantation. The present study provides further evidence on the importance of such characterization by combining speckle tracking echocardiography and innervation imaging.

### Study limitations

There are several limitations that should be acknowledged. First, this was a retrospective, single-centre study. Furthermore, since 2D speckle tracking echocardiography requires good image quality for reliable analysis, selection bias might have been introduced. The patient population was heterogeneous, with ischaemic and non-ischaemic cardiomyopathy patients. Also, ischaemia might have risen during the long follow-up of the study and have influenced the results. Finally, the event rate for appropriate ICD therapy in our population was relatively low compared to previous study (Moss et al. 2004). This might be reflected by the difference in study populations.

## Conclusions

In conclusion, LV GLS measured by 2D speckle tracking echocardiography and H/M ratio measured by planar ^123^I-MIBG SPECT were significantly more impaired in patients who experienced an appropriate ICD therapy compared to patients that did not. In contrast, no differences were observed between groups of patients in terms of LVEF. Multivariate Cox regression analysis showed LV GLS and H/M ratio to be independent predictors of appropriate ICD therapy. These results underscore the importance of multimodality imaging for the characterization of the arrhythmogenic substrate and the identification of patients who will benefit from an ICD.

## Data Availability

Data are available from the corresponding authors on a reasonable request.

## Abbreviations

^123^I-MIBG: ^123^I-meta-iodobenzylguanidine
2D: Two-dimensional
ADMIRE-HF: AdreView Myocardial Imaging for Risk Evaluation in Heart Failure
GLS: Global longitudinal strain
H/M: Heart-to-mediastinum
ICD: Implantable cardioverter defibrillator
IQR: Interquartile range
LV: Left ventricular
LVEDV: Left ventricular end-diastolic volume
LVEF: Left ventricular ejection fraction
LVESV: Left ventricular end-systolic volume
NYHA: New York Heart Association
ROI: Region of interest
SCD: Sudden cardiac death
SPECT: Single photon emission tomography
