# Correction to: Characterization of the left ventricular arrhythmogenic substrate with multimodality imaging: role of innervation imaging and left ventricular global longitudinal strain

**DOI:** 10.1186/s41824-019-0069-z

**Published:** 2019-11-28

**Authors:** Mohammed El Mahdiui, Jeff M. Smit, Alexander R. van Rosendael, Victoria Delgado, Nina Ajmone Marsan, J. Wouter Jukema, Arthur J. H. A. Scholte, Jeroen J. Bax

**Affiliations:** Department of Cardiology, Heart Lung Centre, Leiden University Medical Centre, Albinusdreef 2, 2300 RC Leiden, The Netherlands

**Correction to: Eur J Hybrid Imaging**

**https://doi.org/10.1186/s41824-019-0060-8**

The original publication of this article (El Mahdiui et al., 2019) contained 2 errors that could not be updated prior to publication.

The correct and incorrect information is shown below for clarification. The changed information is shown in bold. These changes do not affect the interpretation and conclusion of the article.

**Page 2**

1. **Incorrect:** Heart failure patients who received an ICD for primary prevention, according to prevailing guidelines (Priori et al. 2015), and who underwent **clinically indicated** a 123I-MIBG…
2. **Correct:** Heart failure patients who received an ICD for primary prevention, according to prevailing guidelines (Priori et al. 2015), and who underwent a 123I-MIBG…

**Page 3**

1. **Incorrect**: For retrospective analysis of clinically acquired data anonymously handled, the institutional review board waived the need for written patient informed consent
2. **Correct**: For retrospective analysis of clinically acquired data anonymously handled, the institutional review board waived the need for written patient informed consent. For a subgroup of patients, the 123I-MIBG scintigraphy was performed under a prospective study (NCT01940081) and the patients provided written informed consent.
